# Clinical Evaluation of Abiraterone in the Treatment of Metastatic Prostate Cancer

**DOI:** 10.4137/CMU.S8337

**Published:** 2013-07-10

**Authors:** Jatinder Goyal, Emmanuel S. Antonarakis

**Affiliations:** 1 Department of Medicine, University of Alabama at Birmingham, Birmingham, AL; 2 Assistant Professor of Oncology, Johns Hopkins Sidney Kimmel Comprehensive Cancer Center, Baltimore MD

**Keywords:** Abiraterone, Androgen receptor, CYP17, prostate cancer

## Abstract

Treatment of castration-resistant prostate cancer remains an area of unmet medical need. Evidence suggests that this entity continues to be driven by androgens and androgen receptor (AR) signaling. Abiraterone acetate, a pregnenolone derivative, is an oral selective and irreversible inhibitor of the key steroidogenic enzyme CYP17. It possesses dual 17-α hydroxylase and C17,20-lyase blocking activity, the result of which is decreased gonadal and extra-gonadal androgen synthesis. Abiraterone was first approved by the US Food and Drug Administration (FDA) in 2011 following the demonstration of superior survival compared with placebo in the post-docetaxel population. Since that time, more evidence has been generated from preclinical studies and clinical trials which have considerably enhanced our understanding of this complex disease. In this paper, we review the development of abiraterone acetate, its pharmacological characteristics, and its effects on the androgen-AR signaling axis, along with the combined experience from clinical trials. We also discuss some of the ongoing trials using this agent, as well as potential mechanisms of abiraterone resistance, novel bio-marker development, and future directions using AR-directed therapies.

## Introduction

Prostate cancer is the second leading cause of cancer deaths in men, with an estimated 29,720 deaths in 2013.^1^ It was clear long ago that the androgen-androgen receptor (AR) axis was essential to the growth and sustenance of prostate cancer. Ever since Huggins and Hodges demonstrated that castration induces remission in prostate cancer patients, androgen deprivation therapy (ADT) has formed the cornerstone of treatment for patients with recurrent and metastatic prostate cancer.^2,3^ However, the overwhelming majority of patients inevitably develop progressive disease on ADT, a state which is often accompanied by elevations in prostate-specific antigen (PSA), which itself is an AR-regulated gene. Formerly termed androgen-independent prostate cancer, this clinically and molecularly heterogeneous tumor state is now more correctly called castration-resistant prostate cancer (CRPC), and is usually a fatal condition.^4^

Patients with CRPC have traditionally been treated with docetaxel as first-line chemotherapy, as this was the first agent to demonstrate improved survival in this disease state.^5^ However, virtually all patients with prostate cancer on docetaxel chemotherapy develop resistance (or intolerance) to it and eventually show progression of disease. In 2010, the US Food and Drug Administration (FDA) approved two new drugs for use in advanced prostate cancer, the autologous immunotherapy product sipuleucel-T and the next-generation taxane agent cabazitaxel.^6,7^ While sipuleucel-T is approved as a first-line agent in asymptomatic or minimally-symptomatic CRPC patients, cabazitaxel is approved as second-line chemotherapy in men who have disease progression on docetaxel treatment.

An increased understanding of the intricacies of the AR pathway eventually resulted in the testing and subsequent FDA-approval of abiraterone acetate in April 2011. The clinical success with abiraterone provided concrete evidence that targeting the androgen-AR axis can lead to improved survival in patients with CRPC.^8^ In this review, we will summarize the knowledge gained from preclinical and clinical studies using abiraterone acetate along with future prospects for this agent. In addition, we have attempted to highlight the impact of abiraterone on our current understanding of the biology of prostate cancer along with questions that have emerged which will need to be addressed in the future.

## Androgen Signaling and its Relevance in CRPC

The AR is a cytoplasmic steroid hormone receptor bound with heat shock protein 90 (HSP 90).^9^ It is composed of a ligand-binding domain at the C-terminus, and a DNA-binding domain at the N-terminus.^10^ When AR is bound by androgen (mainly testosterone and dihydrotestosterone (DHT)), it dissociates from heat shock proteins and translocates into the nucleus where it binds to transcriptional cofactors and androgen response elements (AREs) of target genes that are involved in cell cycle regulation and proliferation.^11^ This androgen-AR axis plays a pivotal role in the pathogenesis of prostate cancer and was intuitively targeted for many years with ADT to stall the uninhibited growth of tumor cells. It is helpful to review the mechanism of action of ADT at this point.

Pulsatile secretion of luteinizing hormone releasing hormone (LHRH) from the hypothalamus leads to luteinizing hormone (LH) and follicle stimulating hormone (FSH) secretion from the anterior pituitary, which in turn are essential for the production of testicular androgens. However, continuous LHRH stimulation using LHRH agonists (e.g., leuprolide, goserelin) leads to desensitization of the LHRH receptor, thereby shutting off LH secretion and hence inhibiting androgen production. This is the rationale for the use of LHRH analogues in patients who develop progressive prostate disease in the form of rising PSA or metastatic disease. Such LHRH analogues have consistently resulted in a significant reduction in circulating testosterone along with tumor shrinkage and relief of symptoms.^12^ However, the vast majority of these patients eventually develop progression of disease (often manifesting as PSA elevations) within 12–36 months of ADT, indicating reactivated AR signaling and often necessitating other forms of treatment including chemotherapy. It is hypothesized that both androgen-sensitive and -resistant phenotypes exist in all prostate cancer patients. Emergence of castration-resistance after androgen withdrawal might therefore be a result of selective propagation of a tumor phenotype that can actively proliferate despite “castrate” levels of serum testosterone (usually defined as ≤ 50 ng/dL).^13^

### AR modulation

Gene expression analysis from human prostate cancer cells has shown that androgen-responsive genes may still be active in patients with castration-resistant disease.^14,15^ Rapidly accumulating data have suggested multiple adaptive mechanisms adopted by tumor cells that could explain the emergence of the castration-resistant phenotype.^12,16^ These include adrenal androgen production despite adequate gonadal suppression, AR upregulation or overexpression,^17^ increase dexpression or function of AR transcriptional coactivators,^18^ increased AR activation by alternative non-canonical pathways including Akt-phosphatidylinositol-3′-(PI3) kinase and mitogen-activated protein (MAP) kinase,^19^ intratumoral androgen synthesis,^20,21^ and the emergence of AR transcriptional variants that lack the ligand-binding domain but retain the constitutively-active N-terminal domain and are ligand-independent. In addition, the AR may undergo mutation resulting in its activation by endogenous steroids, antiandrogens, or medications such as spironolactone.^22^ In sum, a variety of mechanisms ensure the continued dependence of CRPC on androgen stimulation and AR signaling, underpinning the recent surge in enthusiasm for developing drugs that target various steps along the androgen-AR pathway. To this end, abiraterone acetate is the first rationally designed drug that achieves antitumor effects (beyond those achieved with LHRH agonists alone) by suppressing extragonadal and intratumoral androgen synthesis.^23^

### Targeting CYP17 as a novel hormonal therapy

Cytochrome P450 isoform-17 (CYP17) is a microsomal enzyme which coordinates the synthesis of and- rogens and other sex steroids in the adrenal glands and testes by catalyzing two key independently regulated steroidogenic reactions.^24^ Of note, abiraterone acetate was preceded by ketoconazole as a molecular agent to target the extra-gonadal steroidogenic pathway in an attempt to curb CRPC growth.^25,26^ Ketoconazole, which inhibits multiple cytochrome P450 enzymes involved in steroidogenesis (including but not limited to CYP17) was not only found to have marginal antitumor effects in men with CRPC but was also associated with frequent side effects. Ketoconazole was tested in a phase III trial that enrolled 260 patients previously receiving combined androgen blockade and randomized them to ketoconazole in combination with antiandrogen withdrawal (AAWD) versus AAWD alone.^27^ While the ketoconazole arm produced PSA responses in 27% of patients (compared with 11% of patients in the control arm), no survival benefit could be demonstrated with the agent. Moreover, ketoconazole has a complex pharmacology and multiple potential drug-drug interactions, and failed to generate much enthusiasm among prostate cancer oncologists. Finally, ketoconazole requires three-times-per-day dosing as well as corticosteroid supplementation, making its administration a cumbersome task for most patients.^12^

### Pharmacological characteristics of abiraterone

**Figure 1:**
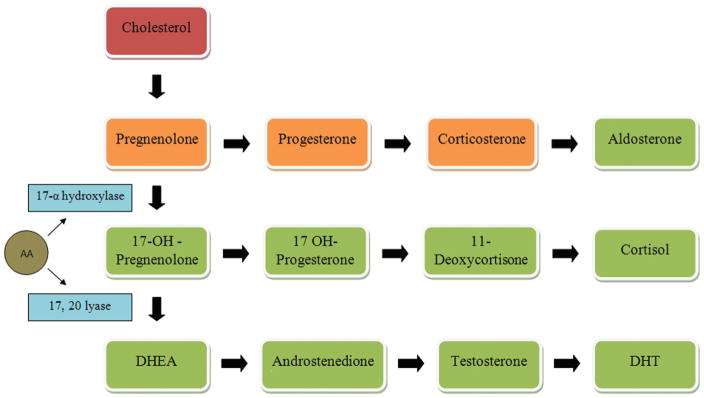
Steroid synthesis pathway with key enzymes essential to abiraterone acetate activity. Abiraterone inhibits 17-α hydroxylase and 17,20 lyase resulting in elevation of steroid hormones depicted in orange, and reduction of hormones depicted in green. **Abbreviations:** AA, Abiraterone acetate; DHEA, dihydroepiandrostenedione; DHT, dihydrotestosterone.

Gaining from the partial successes obtained with ketoconazole, abiraterone acetate was developed at the Institute of Cancer Research, UK, as a potent selective and irreversible inhibitor of CYP17 (Fig. 1). It was a rationally designed agent using a pregnenolone parent structure. Key molecular features (including a 16,17 double-bond and 3-pyridyl substitution) were discovered to be critical for the potent inhibition of CYP17 by abiraterone.^10,28^ This provided a 10 to 30-fold greater inhibitory potential for CYP17 using abiraterone compared to ketoconazole.^29^ Since it selectively inhibits CYP17, it has minimal effects on glucocorticoid biosynthesis (CYP11B1), mineralocorticoid synthesis (CYP11B2), and hepatic drug metabolism (CYP3A4).^30^ However, abiraterone administration is associated with a mild degree of glucocorticoid suppression and a negative feedback-induced rise in adrenocorticotropic hormone (ACTH), thereby resulting in a syndrome of mineralocorticoid excess (Fig. 1). This increased mineralocorticoid production may lead to side effects including hypertension, hypokalemia, and peripheral edema. These effects, however, can be abrogated by introducing low-dose steroids (e.g., prednisone) along with abiraterone, as discussed later in the section on clinical trials.

To increase the bioavailability of abiraterone after oral administration, the 3-β-O-acetate prodrug form of abiraterone was developed, namely abiraterone acetate.^31^ It has a plasma half-life of 10–14 hours and reaches maximum concentrations (C_max_) within 1.5–4 hours after dosing. Abiraterone acetate then undergoes rapid hydrolysis and conversion to the active form in vivo.^32,33^ Phase I studies using the drug also noted that the serum levels varied with concomitant dietary food intake. Investigators found that patients taking high-fat meals had a 4-fold higher exposure than fasting patients, likely due to increased gastrointestinal transit time. Furthermore, variable absorption also contributed to this witnessed discrepancy in serum levels. Since it is difficult to monitor and control dietary intake of individual patients over extended periods of time, the US FDA recommended that abiraterone should be taken on an empty stomach (thereby providing more controlled drug levels). This has generated some controversy within the medical community due to obvious economic implications.^34^ Specifically, if abiraterone is taken with high-fat meals, the milligram amount could be theoretically reduced by approximately 4-fold, resulting in annual savings amounting to thousands of dollars for patients. A clinical trial is currently underway comparing full-dose abiraterone in the fasting and fed states and might help to provide clinical answers to this interesting dilemma (Trial 4 in Table 1; NCT01543776).

## Clinical Experience

### Phase I trials

Phase I dose-escalation studies of abiraterone were carried out to test the safety and efficacy of this oral agent. The first trial treated 21 CRPC patients with once-daily abiraterone acetate in a dose-escalation manner (250, 500, 750, 1000 and 2000 mg) in 3-patient cohorts.^32^ None of these patients had received treatment with ketoconazole. The authors reported that declines in PSA levels of ≥30%, ≥50%, and ≥90% were observed in 66%, 57%, and 29% of patients, respectively. In addition, 62% of patients with confirmed measurable disease had partial responses by Response Evaluation Criteria in Solid Tumors (RECIST) criteria. An increase in ACTH levels and steroids upstream of CYP17 along with a decline in testosterone and downstream androgens and estradiol levels was also documented. Since the endocrine effects of abiraterone reached a plateau at 1000 mg, this dose was chosen for further investigation in phase II and III trials. Side effects in the form of mineralo-corticoid excess (due to feedback ACTH elevation due to partial adrenal corticosteroid synthesis inhibition) manifesting as hypertension, hypokalemia, and extremity edema were seen. These were effectively managed by using a mineralocorticoid receptor antagonist, eplerenone. No grade III or IV dose-related toxicities were seen. This study was therefore successful in proving that selective and continuous inhibition of CYP17 was safe and could also produce durable tumor responses. In addition, the ability of abiraterone to induce PSA responses was potentially important because a >30% decline in PSA had previously been reported to correlate with overall survival in men receiving chemotherapy.^35,36^

In another phase I trial, Ryan et al^33^ recruited 33 patients with progressive CRPC. Nineteen of these patients had received ketoconazole treatment in the past. Abiraterone acetate was orally administered in escalated doses from 250 mg to 1000 mg. A PSA decline of ≥50% at week 12 was seen in 18 of 33 (55%) patients, including 9 of 19 (47%) patients with prior ketoconazole exposure. Furthermore, 7 of 15 (46%) patients who developed ketoconazole-refractory disease demonstrated a response to abiraterone. The high rate of abiraterone responses in these patients suggested the potential superiority of abiraterone over ketoconazole (due to more potent and selective CYP17 inhibition), and at the very least dispelled the notion that ketoconazole-pretreated patients would never respond to abiraterone. A decrease in androgen levels and an elevation in upstream steroid precursors was also documented in this trial. No dose-limiting toxicities were observed. As seen in the previous phase I study, hypertension and hypokalemia were the most commonly seen toxicities but were amenable to medical management. Beta-blockers, diuretics, and eplerenone were used to control hypertension with modest results. This study therefore confirmed the safety and efficacy of abiraterone in men with CRPC, including in patients who had received treatment with prior ketoconazole. The investigators recommended using 1000 mg dose of abiraterone along with addition of low-dose corticosteroid in phase II trials, in order to minimize the mineralocor-ticoid-induced side effects.

### Phase II trials

Due to the favorable results seen in phase I trials, abiraterone acetate was tested in a phase II trial enrolling 58 patients with metastatic CRPC who progressed on docetaxeltherapy.^37^ Prednisone (5 mg twice daily). was given along with 1000 mg of abiraterone daily. The study reported PSA declines of ≥50% in 22 (36%) patients including 7 of the 27 ketoconazole-pretreated patients (26%) at 12 weeks. Overall, confirmed PSA declines of ≥30%, ≥50%, and ≥90%were observed in 47%, 36%, and 16% of patients, respectively. A partial radiographic response evaluated by RECIST criteria was seen in 4 of 22 (18%) patients with evaluable target lesions. The trial also included circulating tumor cell (CTC) conversion rates as an efficacy end point. A conversion from ≥5 to <5 CTCs/7.5 mL after treatment was seen in 10 of 29 (34%) patients. An improvement in the Eastern Cooperative Oncology Group-Performance status (ECOG-PS) was seen in 28% of the patients. Median time to PSA progression was determined to be 24.1 weeks. Most of the witnessed side effects were grade-1 and -2 events, not requiring any additional treatment. The reduced rate of mineralocorticoid-related side effects was attributed to the addition of low-dose prednisone. No patient required treatment with eplerenone on this trial. Although the study showed significant clinical benefit produced by abiraterone, it was reduced in patients who had been treated with ketoconazole, suggesting at least some cross-resistance between ketoconazole and abiraterone.

In a more recent neoadjuvant phase II/pharmacodynamic trial, the combination of leuprolide and abiraterone (30 patients) was tested against leuprolide alone (28 patients) in men with localized high-risk prostate cancer (Gleason score >7).^37a^ The primary aim was to measure and compare intraprostatic testosterone and DHT concentrations. Secondary end points including PSA response, pathologic complete response (pCR), and near pCR (≤5 mm residual tumor). The study showed encouraging preliminary results. Not only was the combination of abiraterone/ leuprolide well tolerated in the neoadjuvant setting, but PSA declines were more frequent and achieved earlier in the abiraterone/leuprolide cohort. The pCR/near pCR rates were higher in the combination arm (34%) than in the leuprolide-alone arm (15%). Finally, intratumoral androgen suppression was more potent and more rapid in the abiraterone/leuprolide cohort. No grade-4 mineralocorticoid-related side effects were seen in the abiraterone group. The study confirmed that intratumoral androgen synthesis is suppressed more potently with abiraterone than with LHRH agonists alone, and also suggested that pCR or near pCR may be a reasonable clinical trial endpoint in the neoadjuvant setting in the context of novel AR-directed agents (although the correlation of pCR with distant metastasis and survival is uncertain).

### Phase III trials

In a landmark trial which eventually led to FDA approval for the drug, abiraterone was tested in a multicenter randomized placebo-controlled study (known as COU-AA-301) in patients with CRPC previously treated with docetaxel.^8^ A total of 1195 men were randomly assigned in a 2:1 ratio to receive prednisone with either abiraterone (797 patients) or placebo (398 patients). The primary end point of the trial was overall survival, while secondary end points included time to PSA progression, progression-free survival, and PSA response rate. The study provided incontrovertible evidence that abiraterone resulted in a higher survival than the control group (14.8 months vs. 10.9 months, *p <* 0.001). To this end, abiraterone/prednisone resulted in a 35.4% reduction in risk of death compared to placebo/prednisone. Furthermore, all secondary endpoints demonstrated a superior response in the abiraterone arm. Abiraterone prolonged time to PSA progression (10.2 vs. 6.6 months, *p <* 0.001), prolonged progression-free survival (5.6 vs. 3.6 months, *p <* 0.001), improved PSA response rate (29% vs. 6%, *p <* 0.001), and objective response rate on the basis of RECIST criteria among patients with measurable disease (14% vs.3%, *p <* 0.0001). Mineralocorticoid-induced adverse effects (hypertension, hypokalemia, and fluid retention) along with cardiac disorders and liver function test abnormalities, were seen at a higher frequency in the abiraterone group. The results of this trial resulted in the FDA-approval of abiraterone plus prednisone for men with docetaxel-pretreated metastatic CRPC.

The updated overall survival analysis of the COU-AA-301 trial was published later by the investigators.^38^ The study ratified the prolongation of overall survival reported on the initial study. At a median follow-up of 20.2 months, median overall survival was longer for the abiraterone group compared to placebo (15.8 vs. 11.2 months, *p <* 0.0001). The treatment effect of abiraterone on overall survival was effective across all prespecified subgroups. The abiraterone group also fared better with respect to time to PSA progression (8.5 vs. 6.6 months, *p <* 0.0001), radiographic progression-free survival (5.6 vs. 3.6 months, *p <* 0.0001), and PSA response rates (29.5% vs. 5.5%, *p <* 0.0001), supporting the initial report of this trial. Although treatment-related adverse effects were similar in both groups, mineralocorticoid-induced adverse events occurred with a higher frequency in the abiraterone cohort. Most commonly seen grade-3 or -4 adverse events included fatigue (9% of men), anemia (8%), back pain (7%), and bone pain (6%).

Following the encouraging results obtained from the COU-AA-301 trial, the role of abiraterone as first-line therapy in chemotherapy-naïve patients was investigated. To examine this issue, abiraterone was then tested in another phase III randomized controlled trial (known as COU-AA-302) in 1088 patients with asymptomatic or mildly symptomatic CRPC disease who had not received previous chemotherapy.^39^ Similarly to preceding trials, prednisone (10 mg daily) was given with 1000 mg of abiraterone (546 patients) or placebo (542 patients) in a randomized fashion. The co-primary end points of this trial were overall survival and radiographic progression-free survival. Due to encouraging results, the study was unblinded and patients were permitted to cross over from placebo to abiraterone after a planned interim analysis which revealed substantial radiographic progression-free survival benefit in the abiraterone group (16.5 months vs. 8.3 months, *p <* 0.001). In addition, overall survival was found to be numerically superior in the abiraterone group at a median follow-up of 22.5 months (median survival not reached vs. 27.2 months placebo, *p* = 0.01). The risk of death was reduced by 25% by the abiraterone-prednisone combination. Although there was a strong trend towards improved survival, it did not cross the O’Brien-Fleming efficacy boundary for statistical significance (*p <* 0.001). Abiraterone also showed superiority over the placebo group with respect to time to first opiate use (median not reached vs. 23.7 months, *p <* 0.001), time to PSA progression (11.1 vs. 5.6 months, *p <* 0.001), time to initiation of chemotherapy (25.2 vs. 16.8 months, *p <* 0.001), and time to decline in ECOG performance status (12.3 vs. 10.9 months, *p* = 0.005). Adverse events in the form of grade-3 and -4 toxicities were marginally higher in the abiraterone group (48% vs. 42%). Liver function test abnormalities and cardiac toxicities were also more common in the abiraterone group. Mineralocorticoid-induced adverse events were higher in the abiraterone group, but they were usually grade-1 or -2 only. The favorable results of the COU-AA-302 trial led the FDA to expand the label for abiraterone to include chemotherapy-naïve patients. Therefore, at the time of writing, abiraterone is currently approved for all men with metastatic CRPC regardless of chemotherapy exposure.

Symptomatic osseous metastatic disease is another hallmark of CRPC and a cause of significant morbidity in patients. The extent of bone metastases and associated pain are indicative of poor survival, as seen in various studies.^40,41^ In an exploratory analysis, the COU-AA-301 investigators subsequently published data on the effect of abiraterone on pain control and skeletal-related events (SREs).^42^ Pain intensity and pain interference with daily activities were assessed using the Brief Pain Inventory-Short Form questionnaire. SREs included pathologic fracture, spinal cord compression, palliative radiation to bone, or bone surgery. In patients with significant pain at baseline (pain scores ≥4/10), abiraterone resulted in greater pain improvement (45% vs. 28.8%, *p* = 0.0005) and faster palliation of pain (5.6 vs. 13.7 months, *p* = 0.0018) compared to placebo. Abiraterone was also superior in terms of reduction of pain interference (60.1% vs. 38%, *p* = 0.0002), time to palliation of pain interference (1 vs. 3.7 months, *p* = 0.0004), and median duration of palliation of pain intensity (4.2 vs. 2.1 months, *p* = 0.0056). Median time to first SREs was also prolonged in the abiraterone group compared to placebo (25.0 vs. 20.3 months, *p* = 0.0001). Thus, the survival benefit obtained from abiraterone (in the post-chemotherapy setting) was supplemented by improved pain relief, delayed time to pain progression, and prevention of SREs, representing a “hat trick” of clinical benefit.^43^

## Abiraterone Resistance

As seen with other agents, including antiandrogen therapies, resistance to abiraterone is a therapeutic concern. Table 2 lists the postulated mechanisms of resistance to abiraterone in treated patients. Although our current understanding of the mechanisms behind abiraterone resistance is still in the nascent stage, pre-clinical and clinical data are beginning to emerge providing vital insights. For example, it is thought that progression of disease on abiraterone therapy is not paralleled by a concurrent increase in androgen levels, providing evidence that the inhibition of CYP17 is irreversible.^33^ However, abiraterone resistance might in part be due to upstream steroid precursors which may activate promiscuous AR.^44,45^ In the phase I trial described above, 4 out of 15 patients who progressed on abiraterone treatment were successfully rescued with dexamethasone, possibly by curbing upstream steroid precursors.^32^

Patients progressing on abiraterone therapy were found to have elevation in PSA, which could be due to ligand-dependent or -independent AR activation.^46^ Proposed mechanisms of abiraterone resistance include intratumoral androgen production,^45^ ligand hypersensitization through AR amplification, transcriptional cofactor dysregulation, and growth factor crosstalk.^47^ Ligand independent mechanisms including activating AR mutations and alternate splice variants have also been proposed.^48^ These AR splice variants lacking the ligand binding C-terminal domain mediate constitutive target gene activation independent of the ligand. Studies in animal models have also shown that tumor relapse on abiraterone was associated with further upregulation of intratumoral CYP17 and other key genes involved in intratumoral androgen synthesis after abiraterone therapy.^45,49^ These include the target gene *CYP17A1*, as well as *CYP11A1*, *AKR1C3*, and *HSD17B3* genes. This study also demonstrated an increase in the expression of full length and truncated AR variants after treatment with abiraterone.^49^ In addition, other parallel pathways have also been implicated in development of resistance to abiraterone. These include the EGFR pathway, Src pathway, and PI3K pathway.^50,51^ These pathways seem to be involved in cross-talk with the androgen-AR pathway thereby affecting tumor cell survival. Activating mutations or overexpressions along these pathways might have an as yet unclearly defined role to play in progression of abiraterone-refractory CRPC.

Taken together, these data do imply that a direct targeting of the AR could potentially overcome abiraterone resistance, at least temporarily.^52^ Enzalutamide is a next-generation direct AR antagonist which might be able to overcome overexpression or amplification of the full-length AR.^53,54^ Alternatively, the introduction of agents that inhibit DHT formation from testosterone (e.g., dutasteride) might also be tested in clinical trials as a way to overcome or delay abiraterone resistance. Additionally, agents such as EPI-001 that target the transcriptionally-active N-terminal domain of the AR, can also potentially be tested and might prove useful particularly in the setting of CRPC driven by AR splice variants.^55^ Other possible targets include AR chaperone proteins such as clusterin,^56^ the dual MET/VEGF-R2 inhibitor cabozantinib,^57^ and the Src kinase inhibitor dasatinib.^50^ Another novel agent, AT13387, an HSP90 inhibitor, is being tested for its efficacy in patients resistant to abiraterone. These and other select ongoing trials have been summarized in Table 1.

## Biomarkers

CRPC is a heterogeneous disease that requires tailored therapy for individual patients based on clinical characteristics specific to each patient. Predicting clinical response (or resistance) to hormonal manipulation a priori through optimal intermediate surrogates is an active area of prostate cancer research. It has been recognized for some time now that changes in PSA levels cannot be used in isolation as a reliable predictor of response to AR-directed therapies. Therefore, there is an unmet medical need to develop novel tumor-specific and treatment-specific biomarkers to accurately assess clinical outcomes and help in developing anticancer therapies.^58^

CTC enumeration is one such promising modality that warrants a closer look. It has been used to prognosticate overall survival in patients with CRPC prior to initiating chemotherapy.^59,60^ In one such study, stratification of patients having a favorable or unfavorable number of CTCs (≤5 or ≥5 per 7.5 mL of blood) was shown to accurately predict survival.^60^ Patients who either remained in the favorable group or converted from unfavorable to favorable category were found to have prolonged survival. The exploration of CTCs as a surrogate marker for response was further substantiated by the COU-AA-301 trial as described above.^60a^ Nevertheless, these efforts currently fall short of validating CTC counts as a surrogate endpoint for survival in men with CRPC, but ongoing phase III studies incorporating CTCs to this end are in progress.

It has been postulated that the TMPRSS2-ERG fusion, which has been identified in about 50% of prostate tumors,^61^ has a role in androgen-dependent tumor growth.^62^ There is evidence to suggest a relationship between TMPRSS2-ERG status and degree of PSA decline in chemotherapy-naïve patients treated with abiraterone, thereby suggesting a possible role as a putative biomarker for abiraterone response.^63^ The authors reported that 12 of 15 (80%) patients with an ERG rearrangement had a PSA decline of at least 90%, whereas only 20 of 62 (32.2%) lacking this rearrangement had such a PSA decline. However, the predictive value of TMPRSS2-ERG could not be verified in another study of 41 men with CRPC receiving treatment with abiraterone in a separate phase II trial.^59^ The gene fusion was found to be present in 15 of the 41 patients (37%) with a median CTC count of 17 per 7.5 mL. The TMPRSS2-ERG fusion was not found to predict a decline in PSA or other clinical outcomes in that analysis. Therefore, the role of TMPRSS2-ERG as a prognostic or predictive factor in men with CRPC remains uncertain at the present time.

Further analysis from the COU-AA-301 study has shown that pretreatment androgen levels (testosterone and its precursors including dehydroepiandrosterone sulfate and androstenedione) as measured by ultrasensitive mass spectrometric assay may be prognostic of overall survival in mCRPC.^63a^ Median overall survival increased in a step-wise fashion per androgen quartile, regardless of treatment (*p <* 0.0001). Baseline androstenedione levels predicting response to abiraterone has also been suggested by some studies.^64,65^ A deeper understanding of the androgen-AR signaling pathway might permit development of more robust biomarkers in the future.

## Unresolved Questions on Abiraterone Therapy

Through the aforementioned trials, it is evident that the androgen-AR axis continues to play a pivotal role in the sustenance and progression of CRPC. In addition, it is also clear that strategically designed drugs can be employed to target some of the key aspects of the pathway to produce clinically desirable effects. By producing improved survival in patients with CRPC, abiraterone has revitalized interest in generating novel agents. However, it has also raised many questions which must be answered over the next several years.

Data from clinical trials supporting the clinical efficacy of abiraterone in both pre-docetaxel and post-docetaxel settings has been generated. However, the optimal sequencing of abiraterone with respect to docetaxel chemotherapy still remains to be elucidated. Advanced prostate cancer has been shown to depend on multiple aberrant pathways.^4^ Therefore, CYP17 inhibition prior to docetaxel therapy could potentially prove to have enhanced antitumor effect.^44^ However, other studies have shown that taxane-mediated anticancer effects might be related in part to their ability to inhibit nuclear transport of AR.^66^ To this end, it is now appreciated that the AR protein associates with microtubules and translocates to the nucleus via the motor protein dynein. Taxanes hyperstabilize microtubules and thereby prevent AR from reaching the nucleus.^67^ This model would predict synergistic antitumor effect by combining taxanes with abiraterone, but would also predict some degree of cross-resistance between taxane agents and abiraterone. However, it is currently unclear how treatment with abiraterone prior to docetaxel would impact response to taxane-based therapy after disease progression down the line. In one such study, the investigators reported that patients who received docetaxel after developing refractoriness to abiraterone showed worse-than-expected responses to docetaxel, providing some evidence of cross-resistance.^68^ These findings require further validation. Finally, the optimal sequencing of abiraterone with other novel agents that have been recently approved (such as enzalutamide) must also be elucidated through well designed trials. Trials of abiraterone in combination with or in sequence with these agents will prove informative in this regard. Patient preference along with physician discretion will therefore play a major role in planning appropriate treatment regimens until more data becomes available.

The use of abiraterone in fasting vs. fed states is another question which is open to debate. With exposure to considerably higher levels of abiraterone in the fed state, the possibility of increased toxicities cannot be ruled out and needs to be investigated further. Conversely, the administration of abiraterone with food might allow the use of lower doses of this agent and would be a more cost-effective option for some patients. Future trials with abiraterone acetate being administered in a fed state could help answer this question (Trial 4 in Table 1; NCT01543776). Another unresolved debate revolves around the duration of therapy. It is unclear whether abiraterone should be administered indefinitely beyond progression (especially in patients who initially respond), a treatment paradigm similar to what is currently employed for LHRH agonists/antagonists. Discontinuing abiraterone and other similar agents could potentially have detrimental effects on the patient, possibly through a flare in testosterone levels. Furthermore, it is unclear if increased doses of abiraterone could potentially overcome resistance to the drug mediated by CYP17 overexpression, though some evidence to suggest this has emerged.^69^ Earlier phase I and II trials had shown that the drug was safe up to a 2000 mg dose, which might justify increasing the dose in future trials to test for abrogation of abiraterone resistance.

An area of considerable interest has been the possible association of ADT with increased risk of cardiovascular disease.^70^ This is conjectured to be due to the effect of low testosterone on increasing insulin resistance, development of metabolic syndrome, and increased overall cardiovascular morbidity and mortality.^71^ Results from the Cancer of the Prostate Strategic Urologic Research Endeavor database showed that ADT was associated with increased mortality from cardiovascular disease in men undergoing treatment for localized prostate cancer.^72^ Similarly, an association of ADT with incident diabetes, incident coronary artery disease, myocardial infarction, sudden cardiac death, and stroke was also reported in an observational study on 14,597 patients treated with ADT.^73^ In the phase III trials described above, abiraterone was also found to be associated with increased cardiovascular toxicity which lends further credit to this hypothesis. However, these cardiac toxicities related more to congestive heart failure as well as tachyarrhythmias rather than cardiovascular complications. Longer follow-up of the COU-AA-301 and COU-AA-302 studies will be required to determine the potential long-term cardiovascular morbidities of abiraterone and if these effects might be additive above and beyond the use of ADT alone.

## Future Targets

There is clear evidence to move abiraterone to an earlier time point in CRPC therapeutics paradigm given its oncologic efficacy and manageable side effects. Based on our enhanced understanding of prostate cancer cell biology, future studies would benefit from combining androgen dependent and non-androgen dependent agents to maximize clinical benefit. Abiraterone can therefore be tested with other chemotherapeutic agents, including recently approved agents in multiple possible combinations on clinical trials. Whether abiraterone can provide incremental benefit when combined with these agents remains to be determined. Enzalutamide is one such agent that has been shown to prolong survival as compared to placebo in men with docetaxel-pretreated CRPC (18.4 months vs. 13.6 months, *p <* 0.0001).^54^ As it directly targets the AR and its nuclear transport, patients at risk of abiraterone resistance could particularly benefit from this agent being used in combination with abiraterone. To this end, a phase III cooperative group trial comparing enzalutamide versus enzalutamide plus abiraterone in chemotherapy-naïve patients is currently being designed, following from an ongoing phase I/II study (Trial 14 in Table 1, NCT01650194).

Combination trials incorporating abiraterone with cabazitaxel, sipuleucel-T, and radium-223 will also be the highlight of the next decade of research. Furthermore, testing of agents that may potentially activate the AR in a ligand-independent manner, including Src kinase (dasatinib), are being investigated in combination with abiraterone.^50^ Dual AR/PI3K pathway inhibition is another lucrative area of clinical exploration.^51^ Data has emerged showing that PI3K inhibition has a net antiproliferative effect and that this effect is synergistic with antiandrogen therapy.^74^ Newer CYP17-targeting agents are also undergoing phase III clinical trials in an attempt to produce more robust clinical responses with more selectivity and less toxicity. Orteronel (TAK-700), a new CYP17 inhibitor with a more selective inhibition of 17,20 lyase over 17-α hydroxylase, has shown encouraging results by lowering androgen levels, reducing PSA levels, and decreasing the levels of CTCs.^75^ Another similar agent, galaterone (TOK-001), which inhibits both CYP17 and AR signaling, also appears promising based on early-phase clinical trials.^76^ However, the perceived advantages of orteronel and galeterone over abiraterone have been questioned by some.

Individualized therapies tailored specifically for the patient will be another focus of investigation in upcoming years. Therapies based on proteomic, genomic, and serologic selection will be an attractive option for CRPC moving forward. Genomic signatures have been developed for AR activity predicting a response to dasatinib.^77^ Future trials that incorporate similar testing for response prediction to abiraterone would be desirable.

## Conclusion

Active research efforts have resulted in an enhanced understanding of the persistent role of androgen and AR in advanced prostate cancer. It had been hypothesized that patients with CRPC continue to be driven by steroid ligands produced downstream of the CYP17 enzyme and that targeting the enzyme through rationally designed drugs could produce clinically beneficial effects. Abiraterone acetate is one such exciting option that has recently appeared on the evolving landscape of treatment options for CRPC. It has not only shown improved overall survival in placebo-controlled randomized trials, but it was also shown to slow down disease progression along with improvement in pain, functionality, and performance status. The clinical success of abiraterone (and more recently, enzalutamide) has proven that the androgen-AR axis is amenable to manipulation and is critical to improve survival in this lethal disease. Toxic effects associated with abiraterone have been consistently found to be grade I or II and are largely related to mineralocorticoid excess. Most of these can be easily abrogated by treatment with a low-dose steroid (e.g., prednisone 5 mg twice daily). Thus, abiraterone confers distinct advantages in terms of toxicity as compared to cytotoxic taxane-based chemotherapy. However, longer follow up is warranted to evaluate long-term toxic effects which might not be immediately apparent such as progressive osteoporosis, metabolic syndrome, and vascular effects.

Like other recently approved agents, while abiraterone may certainly be considered a clinical break through for CRPC, this agent only produces modest improvements in survival and a cure for this disease is still urgently warranted. Primary and acquired resistance to the drug is also a growing concern, which may be abrogated with rational combination strategies. In addition, development of predictive biomarkers guiding individualized therapeutic decision-making remains challenging and is an area of active ongoing research. Although CTC enumeration and TMRPSS2-ERG fusion status have shown some early promise, they need further validation before being universally employed in a clinical setting and they are not surrogates for survival. Although a lot still remains to be discovered, the field of CRPC biology has come a long way from days when no additional therapeutic option existed for patients one decade ago. The current armamentarium is rapidly expanding and promises huge successes in the future towards developing tailored therapy for individual patients.

## Figures and Tables

**Table 1:** Ongoing clinical trials of abiraterone in prostate cancer.

NCT identifier	Trial number	Description	Investigational agent	Mechanism of investigational agent	Status of trial
NCT01576172 (Phase II)	1	Abiraterone acetate and prednisone with or without veliparib in treating patients with metastatic hormone-resistant prostate cancer	Veliparib	PARP inhibitor	Recruiting
NCT01685125 (Phase II)	2	Abiraterone acetate and prednisone with or without dasatinib in treating patients with metastatic hormone-resistant prostate cancer	Dasatinib	TKI	Recruiting
NCT01487863 (Phase II)	3	Concurrent versus sequential treatment with sipuleucel-T and abiraterone in men with metastatic castrate resistant prostate cancer (mCRPC)	Sipuleucel-T	Immunotherapy	Ongoing but not recruiting
NCT01543776 (Phase II)	4	Food effect study of abiraterone acetate for treatment of patients with castration- resistant prostate cancer	–	–	Recruiting
NCT01715285 (Phase III)	5	A study of abiraterone acetate plus low-dose prednisone plus androgen deprivation therapy (ADT) versus ADT alone in newly diagnosed patients with high-risk, metastatic hormone-naive prostate cancer (mHNPC)	–	–	Recruiting
NCT01685268 (Phase I/II)	6	A study of HSP90 inhibitor AT13387 alone or in combination with abiraterone acetate in the treatment of castration-resistant prostate cancer (CRPC) no longer responding to abiraterone	AT13387	HSP90 inhibitor	Recruiting
NCT01637402 (Phase II)	7	A phase II study of increased-dose abiraterone acetate in patients with castration resistant prostate cancer	–	–	Not yet recruiting
NCT01553188 (Phase II)	8	AMG 386 and abiraterone for advanced prostate cancer	AMG 386	Anti- angiogenesis	Recruiting
NCT01681433 (Phase II)	9	OGX-427 in metastatic castrate-resistant prostate cancer with prostate-specific antigen progression while receiving abiraterone	OGX-427	HSP27 inhibitor	Recruiting
NCT01400555 (Phase Ib)	10	A safety study of abiraterone acetate administered in combination with docetaxel in patients with metastatic castration-resistant prostate cancer (mCRPC)	Docetaxel	Cytotoxic chemotherapy	Recruiting
NCT00924469 (Phase II)	11	Neoadjuvant abiraterone acetate plus leuprolide acetate in men with localized high risk prostate cancer	Leuprolide	LHRH agonist	Ongoing but not recruiting
NCT01688492 (Phase II)	12	Combining ipilimumab with abiraterone acetate plus prednisone in chemotherapy and immunotherapy-naïve patients with progressive metastatic castration- resistant prostate cancer	Ipilimumab	Monoclonal antibody (immunotherapy)	Recruiting
NCT01792687 (Phase Ib)	13	Safety, tolerability, pharmacokinetics, and preliminary anti-tumor activity of ascending doses of ARN 509 in combination with abiraterone acetate	ARN 509	Androgen receptor antagonist	Recruiting
NCT01650194 (Phase I/II)	14	A study to determine safety and tolerability of enzalutamide (MDV3100) in combination with abiraterone acetate in bone metastatic castration-resistant prostate cancer patients	Enzalutamide	Androgen receptor antagonist	Recruiting
NCT01511536 (Phase I/II)	15	Cabazitaxel and abiraterone acetate in patients with metastatic castrate-resistant prostate cancer	Cabazitaxel	Cytotoxic chemotherapy	Recruiting
NCT01485861 (Phase Ib/II)	16	Study of GDC-0068 or GDC-0980 with abiraterone acetate versus abiraterone acetate in patients with castration-resistant prostate cancer previously treated with docetaxel chemotherapy	GDC-0068 GDC-0980	PI3K inhibitor	Ongoing but not recruiting
NCT01393730 (Phase II)	17	Abiraterone acetate combined with dutasteride for metastatic castrate resistant prostate cancer	Dutasteride	5-α reductase inhibitor	Ongoing but not recruiting

**Abbreviations:** HSP, heat shock protein; TKI, Tyrosine kinase inhibitor; PARP, poly ADP ribose polymerase; PI3K, Phosphoinositide-3 kinase; VEGFR2, vascular endothelial growth factor receptor 2.

**Table 2:** Postulated mechanisms of resistance to abiraterone in prostate cancer.

Androgen dependent	Intratumoral androgen production Alternative androgen precursors (adrenal or otherwise) Upregulation of key enzymes modulating steroid metabolism
Ligand hypersensitization	AR amplification or overexpression Transcriptional co-factor dysregulation
Ligand independent	AR mutation Alternate splice variants
Alternate pathways	Growth factor cross-talk Interaction with EGFR, Src and PI3K pathways

**Abbreviations:** AR, androgen receptor; EGFR: epidermal growth factor receptor; PI3K, phosphoinositide 3-kinase.
